# SsNEP2 Plays a Role in the Interaction Between *Sclerotinia sclerotiorum* and *Coniothyrium minitans*

**DOI:** 10.3390/jof11020151

**Published:** 2025-02-16

**Authors:** Huizhang Zhao, Zihang Zhu, Yueli Xu, Haixuan Wang, Jiatao Xie, Jiasen Cheng, Daohong Jiang, Yanping Fu

**Affiliations:** 1 Industrial Crops Institute, Hubei Academy of Agricultural Sciences, Wuhan 430064, China; huizhangfungi@hbaas.ac.cn; 2 National Key Laboratory of Agricultural Microbiology, Huazhong Agricultural University, Wuhan 430070, China; zhuzihang@webmail.hzau.edu.cn (Z.Z.); wanghx117@163.com (H.W.); jiataoxie@mail.hzau.edu.cn (J.X.); jiaencheng@mail.hzau.edu.cn (J.C.); daohongjiang@mail.hzau.edu.cn (D.J.); 3 The Provincial Key Laboratory of Plant Pathology of Hubei Province, College of Plant Science and Technology, Huazhong Agricultural University, Wuhan 430070, China; 18202724980@163.com

**Keywords:** *Sclerotinia sclerotiorum*, SsNEP2, necrosis- and ethylene-inducing peptide 1, mycoparasitism

## Abstract

*Sclerotinia sclerotiorum*, a fungal pathogen that is spread worldwide and causes serious diseases on crops, can be parasitized specifically by the mycoparasite *Coniothyrium minitans*. *SsNEP2,* encoding a necrosis-inducing protein in *S. sclerotiorum,* was previously inferred to play a role in the virulence to host plants. In this study, silencing of *SsNEP2* in *S. sclerotiorum* had no significant (*p* < 0.01) influence on mycelial morphology, while overexpression led to lower mycelial growth and more branches. When amended with the fermentation broth of the *SsNEP2* silencing mutants, conidial germination of *C. minitans* was promoted, while conidial production decreased. When parasitized by *C. minitans*, enhanced resistance of the *SsNEP2* silencing mutants and weaker resistance of the overexpressed transformants were observed compared to the wild-type *S. sclerotiorum* strain 1980. In addition, the expression of *SsNEP2* in *C. minitans* enhanced mycelial parasitism on *S. sclerotiorum* and restored the effect of silencing *SsNEP2* in *S. sclerotiorum* on mycoparasitism. Thus, we highlight the role of *SsNEP2* as a PAMP-like protein in the mycoparasitism between *C. minitans* and its host fungus *S. sclerotiorum. SsNEP2* can be used to promote the biological potential of *C. minitans*.

## 1. Introduction

*Sclerotinia sclerotiorum* (Lib.) de Bary is a destructive plant pathogen that is found worldwide on many plants and causes Sclerotinia diseases and enormous economic losses each year [1,2,3,4]. *Coniothyrium minitans* Campbell specifically parasitizes mycelia and sclerotia of *S. sclerotiorum* and reduces sclerotia inocula in soil. Therefore, it has been developed into a commercial microbial agent to control Sclerotinia diseases [5,6]. Though the mycoparasitic mechanisms between *S. sclerotiorum* and *C. minitans* have received much attention, most of the research has focused on *C. minitans* [7,8,9,10]. The response of the host *S. sclerotiorum* to parasitism is largely undiscovered [11].

Necrosis- and ethylene-inducing peptide 1 (NEP1) from the culture filtrates of *Fusarium oxysporum*, a pathogen to *Erythroxylum coca*, was the first isolated NEP1-like protein (NLP) [12,13]. Since then, NLPs have been identified as ubiquitous in bacteria, oomycetes, and fungi, while no NLP homologs are found in animals, plants, or archaea [14,15,16,17,18,19]. NLP critically contributes to the virulence of fungal pathogens and the spread of diseases. The NLP gene copy number in different species varies. The largest NLP family, encoded by 50-60 loci in each species, was discovered in the *Phytophthora* species [20]. *Neurospora crassa* carries a single gene encoding NLP. *Magnaporthe oryzae* and *Gibberella zeae* each contain four NLP genes [21]. Two NLP homologs are present in *S. sclerotiorum* and its closely related genus *Botrytis* [22,23]. There are also two NLPs predicted in the genome of two *Sclerotinia* parasitic fungi: *Trichoderma harzianum* [24] and *Clonostachys rosea* [25]. All NLPs share a conserved heptapeptide motif “GHRHDWE” and either two or four conserved cysteine residues [26]. Based on the cysteine residues and phylogenetic analysis, NLPs are divided into two types: type I, which contains two cysteine residues, and type II, which has four cysteine residues [16]. Both types are cytotoxic to plant cells, albeit with different mechanisms. While type I NLPs possess a cation-binding pocket, which is required for cytotoxicity, type II NLPs contain a cytotoxicity-associated calcium-binding motif [17].

Up-regulation of *NLP* is an important phenomenon in pathogens during the infection process [27,28]. In *M. oryzae*, all four *MoNLPs* were found to be significantly up-regulated during the infection of rice plants, and the quadruple *ΔMoNLP* mutants showed similar virulence, sporulation, and radial growth under various unfavorable cultural conditions to the wild-type strain P131, indicating that the Nep1-like protein family is dispensable for infection to rice plants and itself [21]. Both *VdNLP1* and *VdNLP2* (both belonging to type I) of *Verticillium dahliae* were highly induced at the late stage of infection of *Solanum lycopersicum* leaves, while deletion of them had no significant influence on radial growth and germination of the conidiospores in *V. dahlia* [29]. Deletion mutants of *BeNEP1* and *BeNEP2* were morphologically indistinguishable from the wild-type strain *B. elliptica*, and similar disease symptoms and radial lesion sizes were also observed [28]. Both *BcNEP1 and BcNEP2* were differently expressed during the infection of *Nicotiana benthamiana* leaves, and equal virulence to the wild-type *B. cinerea* was observed in *BcNEP2* or *BcNEP1* replacement mutants [30]. These findings suggest that NLPs are not directly the pathogenic determinant in these plant–pathogen interaction systems.

In *S. sclerotiorum* strain 1980 (NCBI txid: 665079), there are two type I NLP-encoding genes, namely *SsNEP2* (GenBank: XM_001586833) and *SsNEP1* (GenBank: XM_001596807). Both of them contain a conserved domain of the necrosis-inducing protein (NPP1, pfam05630), which possesses a conserved heptapeptide “GHRHDWE” and one predicted disulfide bond formatted with two conserved cysteine residues. As effector candidates, they are predicted to be pathogen-associated molecular patterns (PAMPs) and associated with the broad host range of *S. sclerotiorum* [31,32]. During infection of *Brassica napus* leaves, *SsNEP1* was found to be expressed at a very low level compared to *SsNEP2* [23]. *SsNEP2* contributes to the virulence of *S. sclerotiorum* by regulating reactive oxygen species (ROS) without affecting the development of mycelia [33]. In the mycoparasitic process between *C. minitans* and *S. sclerotiourm*, we found that *SsNEP2* was also significantly induced 12 h post-inoculation (hpi) when in contact with *C. minitans*, while the expression of *SsNEP1* and *NLPs* in *C. minitans* was not regulated [11]. *C. minitans* specifically parasitizes *S. sclerotiorum*, but not *B. cinerea*. Based on RNA-seq and the qRT-PCR analysis, *NLPs* were found to be slightly up-regulated or undetectable during the interaction between *B. cinerea* and *C. minitans* [34]. Thus, it is postulated that *SsNEP2* may play an additional role in the mycoparasitic process besides inducing necrosis on plant cells. In this research, the function of *SsNEP2* in the mycoparasitism between *C. minitans* and *S. sclerotiorum* was investigated.

## 2. Materials and Methods

### 2.1. Strains and Growth Conditions

*C. minitans* strain ZS-1 was originally isolated from soil in Zhushan County, China. Strain ZS-1 was used to activate the response of *S. sclerotiorum* 1980. Fungal strains were grown on potato dextrose agar plates (PDA, DB Biosciences, San Diego, CA, USA) at 20 °C and stored in PDA slants at 4 °C for further use [35]. Bacteria used for molecular experiments were grown on Luria–Bertani medium.

### 2.2. RNA Preparation and cDNA Synthesis

Mycelia of strain 1980 were cultured on a sterile cellophane membrane on PDA for 48 h. Conidia of *C. minitans* strain ZS-1 were shaken at 20 °C in PDB at 150 r/min for 36 h, washed with sterilized water 3 times, and re-suspended in water to 1.0 × 10^6^ conidia/mL. Then, the suspension with germinated conidia was spread on the colony (1 mL for each plate) of *S. sclerotiorum*. The mycelial mixtures were sampled at 0 h (immediately after spread), 4 h (co-cultured for 4 h), 12 h, 24 h, 48 h, 72 h, and 96 h. Similarly, mycelia of *S. sclerotiorum* cultured for the same duration as the mixed sample were sampled at 48 h, 52 h, 60 h, 72 h, 96 h, 120 h, and 144 h. For all the samples, total RNA was extracted using RNA reagent (NewBio Industry, Tianjin, China) following the manufacturer’s instructions. To check the gene expression at different interaction time points, cDNA synthesis was conducted according to the manufacturer’s protocol using *EasyScript*^®^ cDNA synthesis kits (TransGen biotechnology, Beijing, China).

### 2.3. Yeast Secretion Trap Screen Assay

The predicted signal peptide fragment of *SsNEP2* was fused to the N-terminus of the secretion-defective invertase gene (*suc2*) in the vector pSUC2 and then transformed into the yeast strain YTK12 using the LiAc/SS carrier DNA/PEG method [36,37]. Yeast candidates were screened and cultured on CMD-W medium lacking tryptophan (0.67% yeast N base without amino acid, 0.075% W dropout supplement, 2% sucrose, 0.1% glucose, and 2% agar) and YPRAA (10 g/L yeast extract, 20 g/L peptone, 20 g/L raffinose, 2 mg/L antimycin A, and 2% agar) medium; strains with secretion activity were able to grow on YPRAA. The secretion sucrase activity of the candidates was assayed using 2,3,5-triphenyltetrazolium chloride (TTC). The supernatant was centrifuged and collected after the candidate transformants were incubated in a 10% sucrose solution at 30 °C for 35 min. Then, the final concentration of 0.1% TTC reagent was added and stewed at room temperature for 5 min to observe the color change in the test tube. A positive reaction changed from colorless to dark red, with the SP^Avrb^ transformant, pUSC2 transformant, and YTK12 strains used as positive and negative controls, respectively.

### 2.4. Vector Construction and Fungal Transformation

Plasmid pCH, with a hygromycin phosphotransferase gene (hyg) as a marker, was used to construct the *SsNEP2* RNAi vector. Sense and antisense fragments, which were repeats of a 500 bp fragment, were amplified using two primer pairs (Table 1) with full-length cDNA of *SsNEP2* as the template. For the sense fragment, primers *SsNEP2*-*Sma*I F and *SsNEP2*-*Bam*HI R were used. Primers *SsNEP2*-*Cla*I F and *SsNEP2*-*Eco*RV R were used to amplify the antisense fragment. The primers were designed based on the target segment using the software Primer Premier 6.0 (PREMIER Biosoft, Palo Alto, CA, USA), and restriction enzyme sequences were added at each end. cDNA was synthesized from 5 μg of total RNA of every sample using oligo(d)T in the EasyScript One-Step gDNA Removal and cDNA Synthesis SuperMix Kit (TransGen biotechnology, Beijing, China). PCR amplification was conducted with the Pfu DNA Polymerase Kit (TransGen Biotechnology, Beijing, China), and the program was as follows: 94 °C for 5 min, 94 °C for 30 s, 56 °C for 15 s, and 72 °C for 15 s, repeats of 34 cycles from the second step, followed by a final extension at 72 °C for 10 min. The fragments were cloned into a pCIT vector with a spacer fragment to make a hairpin RNAi structure. Then, the recombinant silencing sequence with a promoter and a terminator was cut using a pair of restriction enzymes, *Xho*I and *Sac*I, and introduced into a pCH vector to construct pCH-*SsNEP2*. pCH-*SsNEP2* was transferred into *Agrobacterium tumefaciens* EHA105 and used to transform fungal mycelia through *A. tumefaciens*-mediated transformation (ATMT), which was performed as described previously [38]. The transformants were selected on PDA with 100 μg/mL hygromycin B (Sigma-Aldrich, Shanghai, China).

To overexpress *SsNEP2* in *C. minitans* and *S. sclerotiorum*, pCETNSF-*SsNEP2* was constructed with the full-length *SsNEP2* CDS fragment and an *EF-1α* promoter and was introduced into fungal protoplasts with the help of polyethyleneglycol. The transformants were selected on PDA with a final concentration of 100 μg/mL G418 (Sigma-Aldrich, Shanghai, China). The full-length *SsNEP2* CDS fragment was obtained with the primer pairs OEs*sNEP2*-SpeI F and OEs*sNEP2*-KpnI R. Fungal protoplasts were prepared and transformed as described by Rollins [39] and Qiao et al. [40] with modifications. Fungal protoplasts were generated in 0.8 mol/L MgSO_4_ lysis solution buffer containing 1% lysing enzymes from *T. harzianum* (Sigma-Aldrich, Shanghai, China) and 0.1% Snailase (Sigma-Aldrich, Shanghai, China) and re-suspended in STC (1.0 mol/L sorbitol, 0.05 mol/L Tris-HCl pH 8.0, and 0.05 mol/L CaCl_2_) at a concentration of 1.0 × 10^8^ per milliliter.

The relative expression of *SsNEP2* in transformants was determined as described below using RT-qPCR with the primer pairs Rt*SsNep*2-F/R and normalized under the primer pairs *CmActin* F/R in *C. minitans* and *β-tubulin* F/R in *S. sclerotiorum* (Table 1).

### 2.5. RT-qPCR

RT-PCR (reverse transcription PCR) was used to detect the gene expression of *SsNEP1* and *SsNEP2* at different stages. PCR was conducted with 0.2 μL cDNA for each sample and under an annealing temperature of 56 °C using a Bio-Rad thermal cycler (Bio-Rad, Hercules, CA, USA). The PCR products were detected with 1.5% agarose electrophoresis and imaged under UV light. The PCR product of *S. sclerotiorum β-tubulin* was used to sterilize the total cDNA. Two independent repeats of the experiment were performed.

The relative transcripts of the NPP1-related gene and silencing efficiency of *SsNEP2* were determined by RT-qPCR (quantitative reverse transcription PCR) on a Bio-Rad CFX Real-Time System (Bio-Rad, Hercules, CA, USA). Each PCR reaction contained 7.5 μL of 2×iTaq Universal SYBR Green Supermix (Bio-Rad, Hercules, CA, USA), 0.2 μL of cDNA template, 0.3 μL of each primer, and 6.7 μL of ddH_2_O (double distilled water). The program was as follows: 95 °C for 2 min, followed by 42 cycles of 95 °C for 15 s, 57 °C for 15 s and 72 °C for 15 s, and a cycle with 0.5 °C per second from 65 °C to 95 °C to remove the influence of the primer dimer. Total cDNA abundance in the samples was normalized against *S. sclerotiorum β-tubulin*. The primers used to obtain an amplicon of approximately 150 bp from each target gene are listed in Table 1. The relative expression of *NLPs* in *S. sclerotiorum* was conducted with the primer pairs Rt*SsNep*2-F/R for *SsNEP2* and the primer pairs Rt*SsNep1*-F/R for *SsNEP1*. Relative gene expression was normalized under the primer pairs *β-tubulin* F/R. The primer pairs for RT-PCR and RT-qPCR were designed by the software Beacon Designer 7.9 (PREMIER Biosoft, Palo Alto, CA, USA). All samples were amplified in triplicate.

### 2.6. Mycelial Growth Assay

Mycelial growth assay was performed on PDA plates as described by Cheng et al. [41]. Strains were activated on PDA plates under 20 °C. Mycelial agar disks were taken from the colony margin with a sterilized hole punch (Φ = 5 mm), transferred face down to the center of fresh 20 mL PDA plates (Φ = 90 mm), and incubated at 20 °C. For *S. sclerotiorum*, the mycelial diameters were measured using a centimeter ruler every 12 h after inoculation and converted to daily data. The mycelial growth of *C. minitans* was measured every day after inoculation from the 3rd day to the 5th day. Mean value was taken to calculate the mycelial growth rate in centimeters every day (cm/d). Each treatment contained three replicates, and the entire experiment was repeated three times.

### 2.7. Parasitic Test on Mycelia and Sclerotia of S. sclerotiorum by C. minitans

The dual-culture method was used to determine the mycoparasitic ability of *C. minitans* to the mycelia of *S. sclerotiorum* [9]. *S. sclerotiorum* was placed on one side of the PDA plate (Φ = 90 mm), and *C. minitans* was placed on the opposite side of the same plate. The dual-culture plates were incubated for 30 days. To quantify the parasitic ability of the mutants, from the junction of the two fungi, the *S. sclerotiorum* colony was divided into four regions, and five disks from each region were punched and placed on fresh PDA plates. After seven days of incubation, the emerging colonies were judged to be those of *C. minitans* or *S. sclerotiorum*.

Parasitization of sclerotia was evaluated following the description given in [41]. Sclerotia were cultured on carrot blocks in 500 mL flasks for 30 days. Sclerotia with similar size were selected to test the parasitic ability of *C. minitans*. Conidia of *C. minitans* were collected from colonies growing on PDA for 15 days and diluted with sterilized ddH_2_O to 1.0 × 10^6^ conidia/mL. Sclerotia were submerged in 50% bleach twice for 5 min to remove the microorganisms on the surface, rinsed in 70% ethanol twice for 5 min, and washed with sterilized ddH_2_O thrice. The surface-sterilized sclerotia were submerged into the conidial suspension of *C. minitans* for 30 min, half-buried in sterilized wet sand in plates, and incubated for 30 days at 20 °C. Sclerotia were taken out from the sand and dissected using a surgical knife to observe the cross-section using a stereomicroscope (Olympus SZX-16, Olympus Corporation, Tokyo, Japan). Twenty sclerotia with five repeats were used for each treatment, and the experiments were repeated twice independently.

### 2.8. Conidial Production of C. minitans Induced by S. sclerotiorum Culture Filtrate

To clarify the influence of *SsNEP2* on *C. minitans* conidial production, 10 mycelial discs (Φ = 5 mm) of *S. sclerotiorum* were transferred to 500 mL Erlenmeyer flasks with 200 mL PDB (DB Biosciences, San Diego, CA, USA) and incubated at 150 r/min under 20 °C for four days. Then, the cultures were subjected to centrifuge at 5000 r/min for 15 min at 4 °C, and all hyphal fragments were removed by passing through a sterile 0.22 μm filter (Millipore Corporation, Danvers, MA, USA). The filtered liquid (PDB fermentation broth, FPDB) was stored in sterile tubes and buried in ice for future use. The FPDB was amended with PDA or water containing 2% agar (WA) at a 1:1 volume to make PDA-FPDB or WA-FPDB, respectively. A mycelial agar (Φ = 5 mm) of *C. minitans* strain ZS-1 from a 5-day colony on PDA was placed in the center of the plate with 8 mL of PDA-FPDB or WA-FPDB and incubated at 20 °C. The mycelial growth was measured using the cross method every 24 h from 48 h to 120 h. The experiment was repeated thrice independently.

Conidia of *C. minitans* ZS-1 formed were calculated after 15 days as follows. Four mycelial agars (Φ = 5 mm) of *C. minitans* were triturated and filtered with a layer of sterilized lens paper. Then, the filtered liquid was centrifuged at 12,000 r/min for 5 min to remove the liquid supernatant. Finally, the precipitate was re-suspended with sterilized water to 1 mL, and conidia were calculated using a hemocytometer (Qiujing XB.K.25., Shanghai, China). Data were logarithmically transformed and used for statistical analysis.

### 2.9. Conidial Germination of C. minitans Induced by S. sclerotiorum

To determine the promotion of conidial germination by the sclerotia silencing mutants, the sclerotial soak was prepared for the germination of *C. minitans* conidia at 20 °C. Firstly, 50 g of sclerotia from the *SsNEP2* silencing mutants or the wild-type strain 1980 were sterilized as described above in method Section 2.7 and incubated in 50 mL sterilized water for 24 h at 20 °C. Then, the mixed solution was centrifuged at 5000 r/min under 4 °C for 10 min, and the supernatant was filtered through a 0.22 μm filter and used to dilute the conidia of *C. minitans* to 10^5^ conidia/mL. The conidia were shaken at 150 r/min under 20 °C for 36 h to check the germination with a light microscope. The sterilized water was used as the negative control, and all the trials were conducted with four repeats for each treatment.

### 2.10. Statistical Analysis

The significant value of the differences in all analyses was evaluated using the least significant difference test with the ANOVA program in the SAS 9.2 software (SAS Institute, Cary, NC, USA) at the significance level of *p* < 0.01.

## 3. Results

### 3.1. SsNEP2 Is Highly Expressed During the Mycoparasitic Process

**Figure 1:**
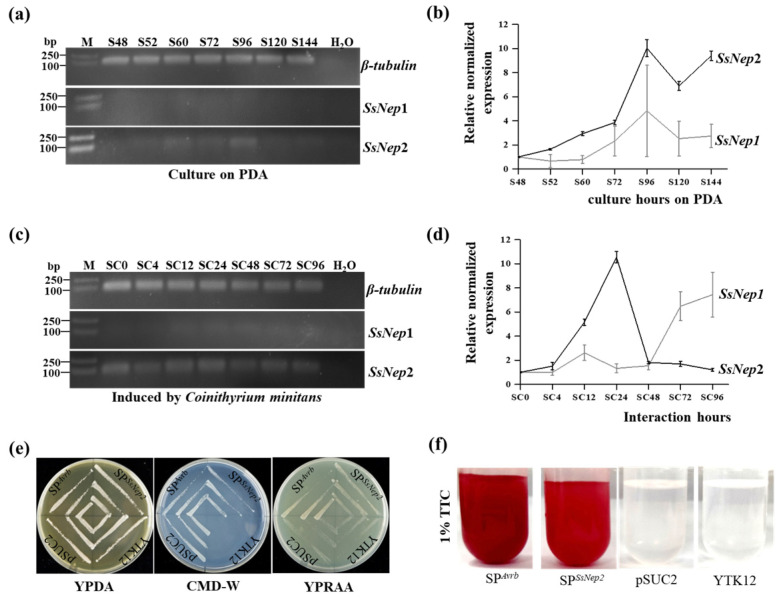
Expression of *SsNEP1* and *SsNEP2* in *S. sclerotiorum*. (**a**) RT-PCR detection of the gene expression of *SsNEP1* and *SsNEP2* in *S. sclerotiorum* cultured on PDA. S48, S52, S60, S72, S96, S120, and S144 represent the incubating stage of *S. sclerotiorum* on PDA for 48 h, 52 h, 60 h, 72 h, 96 h, 120 h, and 144 h, respectively. *β-tubulin* was used as a reference. H_2_O was used as the negative control. M: DNA ladder DL2000 (TaKaRa, Dalian, China). The primer pairs were Rt-SsNEP2-F/R for *SsNEP2* and Rt-SsNEP1-F/R for *SsNEP1*. The primer sequences are listed in Table 1. (**b**) Fluorescence quantitative PCR detection of gene expression of *SsNEP1* and *SsNEP2* in *S. sclerotiorum* cultured on PDA. The normalized gene expression at 48 h was set as 1. The expression of *β-tubulin* was used to normalize different samples. (**c**) The expression of *SsNEP1* and *SsNEP2* in *S. sclerotiorum* induced by *C. minitans* at different stages. SC0, SC4, SC12, SC24, SC48, SC72, and SC96 represent the interaction stages of *S. sclerotiorum* with *C. minitans* for 0 h, 4 h, 12 h, 24 h, 48 h, 72 h, and 96 h, respectively. (**d**) The transcript level of *SsNEP1* and *SsNEP2* in *S. sclerotiorum* induced by *C. minitans* at different stages. The normalized gene expression at 0 h was set as 1. (**e**) Secretory identification of SsNEP2 using the yeast secretion trap system. SP^Avrb^ is the positive control. pSUC2 is the empty vector control. YTK12 is the yeast control. (**f**) Color identification of the samples from (**e**) by TTC. TTC is 2,3,5-triphenyltetrazolium chloride. TTC changed from colorless to red, indicating a responsive activity.

The expression of *SsNEP1* and *SsNEP2* was examined during the interaction between *S. sclerotiorum* and *C. minitans*. *SsNEP2* was expressed at a very low level in *S. sclerotiorum* cultured on PDA*,* while it was strongly induced at 12 hpi and 24 hpi when parasitized by *C. minitans*, and the highest transcript level was detected at 24 hpi. Both during the mycoparasitic process and during culturing on PDA, *SsNEP1* was expressed at a relatively lower level compared to *SsNEP2* (Figure 1a–d). The results suggest that *SsNEP2*, not *SsNEP1*, may be involved in the interaction between *S. sclerotiorum* and *C. minitans*. Therefore, SsNEP1 was chosen to carry out further study.

*SsNEP2* is predicted to encode a secreted signal peptide at the N-terminus. The secretory activity was identified based on a yeast secretion trap system (Figure 1e). Yeast containing the *SsNEP2* plasmid SP^SsNep2^ could degrade sucrose, causing TTC to change from colorless to red, indicating the secretory activity of the signal peptide (Figure 1f).

### 3.2. Silencing of SsNEP2 Has No Influence on the Growth of S. sclerotiorum

**Figure 2:**
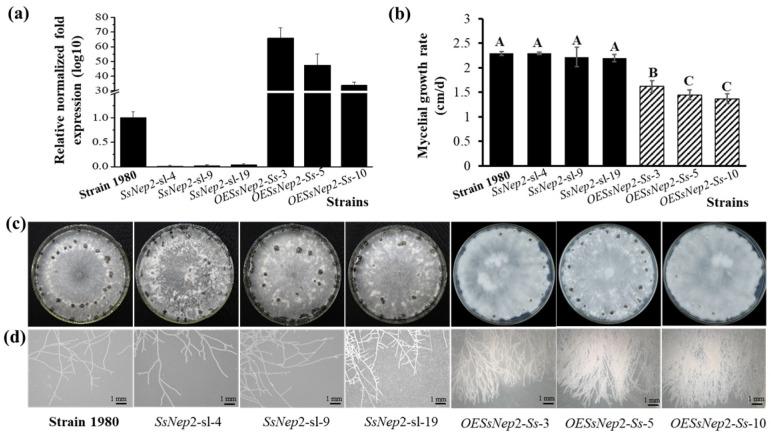
*SsNEP2* mutants of *S. sclerotiorum*. (**a**) The transcript level of *SsNEP2* cultured for 36 h on PDA. Three silencing mutants are represented as *SsNEP2*-sl-4, *SsNEP2*-sl-9, and *SsNEP2*-sl-19. OE*SsNep2*-Ss-3, OE*SsNep2*-Ss-5, and OE*SsNep2*-Ss-10 represent the overexpressed transformants of *S. sclerotiorum*. (**b**) Radial growth of all the strains on PDA. The significance level is *p* < 0.01 and the significant differences are distinguished by capital letters of A, B and C at the top of bar chart. (**c**) Colony morphology of all the strains cultured on PDA for 7 days at 20 °C. (**d**) Hyphal tips of all strains growing on PDA for 36 h.

Three silencing mutants (*SsNEP2*-sl-4, *SsNEP2*-sl-9, and *SsNEP2*-sl-19) and three overexpressed transformants (OE*SsNep2*-*Ss*-3, OE*SsNep2*-*Ss*-5, and OE*SsNep2*-*Ss*-10) of *S. sclerotiorum* were obtained and confirmed by RT-qPCR (Figure 2a). Compared to the wild-type strain 1980, the silencing mutants displayed similar colony morphology, growth rate, hyphal tips, and sclerotial formation, while the overexpressed transformants grew much more slowly with more branches (Figure 2b). Overexpression of *SsNEP2* in *S. sclerotiorum* led to irregular colonies and fewer and smaller sclerotia compared to strain 1980 (Figure 2c,d). The results suggest that silencing of *SsNEP2* has no influence on the growth of *S. sclerotiorum*, while overexpression of *SsNEP2* leads to significant inhibition of mycelial growth with more branches of mycelia.

### 3.3. Silencing of SsNEP2 in S. sclerotiorum Inhibits the Conidial Production of C. minitans

**Figure 3:**
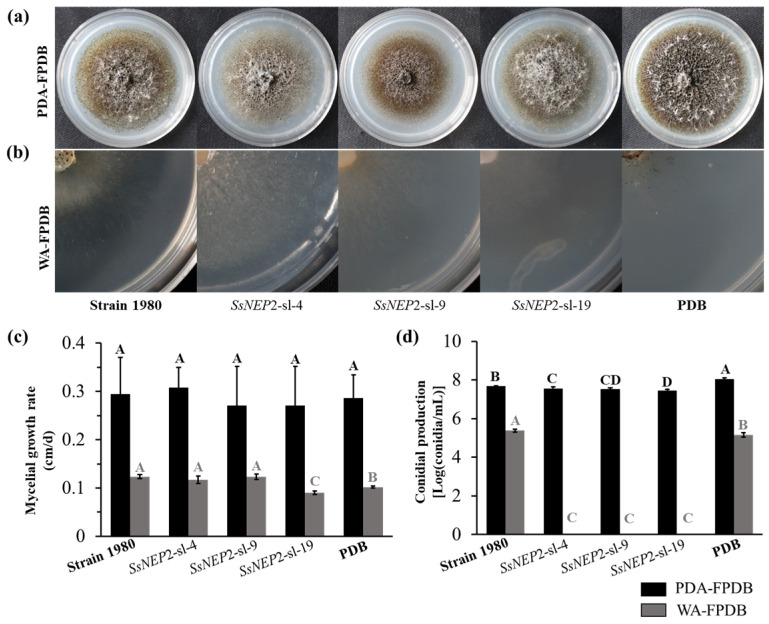
*C. minitans* affected by *SsNEP2*. (**a**) Colony morphology of *C. minitans* strain ZS-1 incubated at 20 °C for 15 days on PDA-FPDB (PDA amended with an equal volume of filter fermentation broth of *S. sclerotiorum* shaken in PDB for four days). (**b**) Colony morphology of *C. minitans* strain ZS-1 incubated at 20 °C for 15 days on WA-FPDB (water agar culture amended with filter fermentation broth of *S. sclerotiorum*). (**c**) Mycelial growth of *C. minitans* on PDA-FPDB and WA-FPDB. The significance level is *p* < 0.01 and the significant differences are distinguished by capital letters of A, B and C at the top of bar chart. (**d**) Conidiation of *C. minitans* cultured on PDA-FPDB or WA-FPDB with 15 days. The significance level is *p* < 0.01 and the significant differences are distinguished by a capital letter at the top of bar chart.

To evaluate the influence of *S. sclerotiorum* on *C. minitans*, strain ZS-1 was incubated on PDA-FPDB (PDA amended with fermentation broth of *S. sclerotiorum*) and WA-FPDB (water agar amended with fermentation broth of *S. sclerotiorum*) (Figure 3a,b). An average mycelial growth rate of 0.09 cm/d was observed when cultured on WA-PDA of *SsNEP2*-sl-19, which was significantly slower than on PDB negative control and other treatments. No significant influence on the mycelial growth of strain ZS-1 was detected on PDA-FPDB (Figure 3c).

Approximately 3.75 × 10^7^, 3.48 × 10^7^, and 2.85 × 10^7^ conidia/mL were produced when strain ZS-1 was incubated on PDA-FPDB of *SsNEP2*-sl-4, *SsNEP2*-sl-9, and *SsNEP2*-sl-19, respectively, which were fewer than those produced on the PDA-FPDB of strain 1980 (4.73 × 10^7^ conidia/mL) and the negative control of PDB (1.15 × 10^8^ conidia/mL) (Figure 3d). However, no conidium was formed when strain ZS-1 was incubated on WA-FPDB of all *SsNEP2* silencing mutants (Figure 3d). Thus, the expression of *SsNEP2* is essential for the conidiation of *C. minitans* during the mycoparasitic process.

### 3.4. Silencing of SsNEP2 in S. sclerotiorum Enhances Resistance to the Parasitism of C. minitans

**Figure 4:**
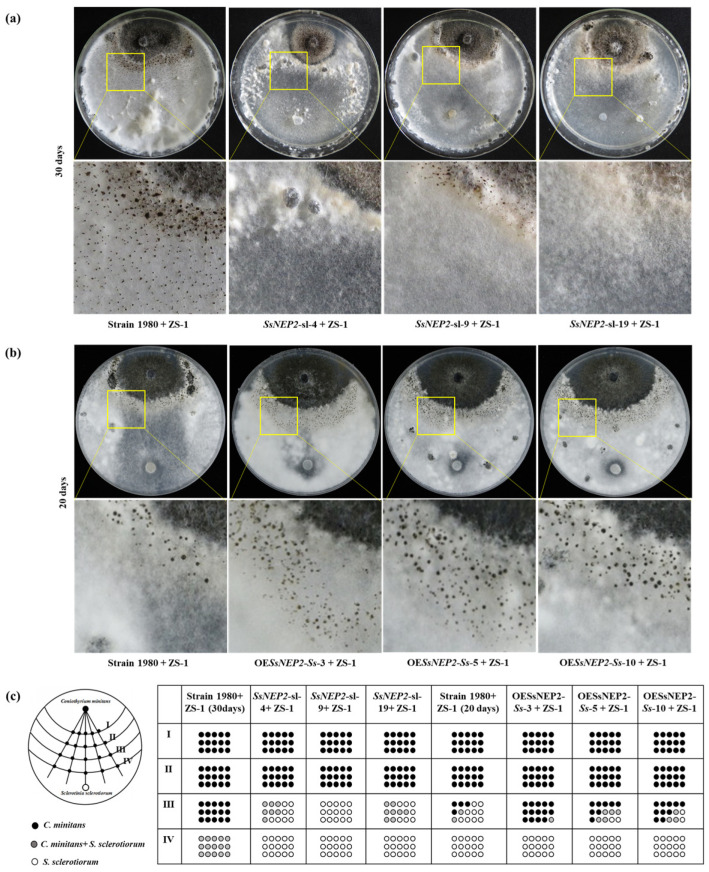
Mycelial parasitism of *C. minitans* against the *SsNEP2* mutants of *S. sclerotiorum.* (**a**) Dual culture of *C. minitans* and the *SsNEP2* silencing mutants for 30 days. ZS-1 means *C. minitans.* (**b**) Dual culture of *C. minitans* and the *SsNEP2* overexpressed transformants for 20 days. (**c**) Schematic diagram showing the number of agar disks that gave rise to either *C. minitans* (black circle), *S. sclerotiorum* (hollow circle), or both (gray circle). Each circle represents a colony developed from a mycelial agar disk sampled from zones I, II, III, or IV between the inoculation sites of *C. minitans* and *S. sclerotiorum* in a dual culture. The appearance of *S. sclerotiorum* colonies indicates that *C. minitans* has not yet parasitized *S. sclerotiorum* in this region. Similarly, the presence of *C. minitans* colonies suggests that *S. sclerotiorum* was eliminated by *C. minitans*. The occurrence of colonies of both *C. minitans* and *S. sclerotiorum* indicates that *C. minitans* has not yet completely destroyed the hypha of *S. sclerotiorum*.

To clarify the role of *SsNEP2* in the mycoparasitism between *S. sclerotiorum* and *C. minitans*, the mycoparasitic ability of ZS-1 on the *SsNEP2* transformants was evaluated by dual culture. Compared to the parasitism on *S. sclerotiorum* 1980, strain ZS-1 exhibited significantly weaker parasitic ability on silencing mutants and formed fewer pycnidia, while stronger parasitism and more pycnidia were observed on the colony of the overexpressed transformants of *S. sclerotiorum* (Figure 4).

**Figure 5:**
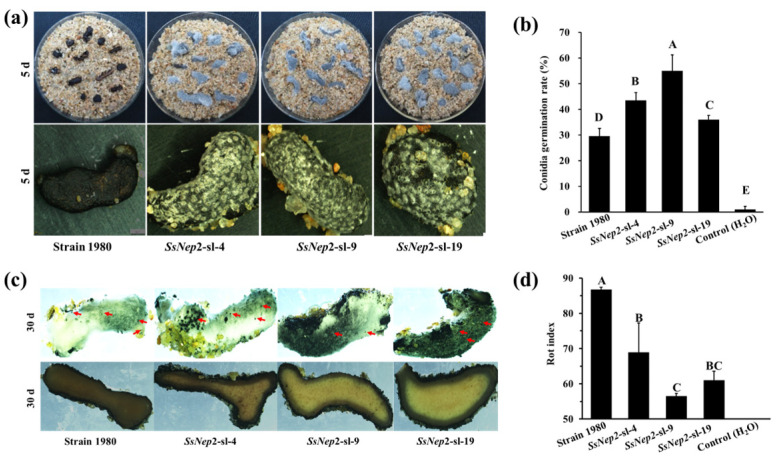
Parasitism of *C. minitans* to the sclerotia of *S. sclerotiorum*. (**a**) Sclerotia of *SsNEP2* silencing mutants were covered with mycelia of *C. minitans* after inoculation with *C. minitans* conidia for 5 days at 20 °C. Strain 1980 is *S. sclerotiorum* strain 1980 (**b**) The conidial germination rate of *C. minitans* cultured in sclerotial soaked for 24 h at 20 °C. (**c**) Sclerotia of *S. sclerotiorum* parasitized by *C. minitans* 30 days after inoculation. Pycnidia formed on the surface of sclerotia (red arrows). (**d**) Rot index of sclerotia of the silencing *SsNEP2* mutants parasitized by *C. minitans*. The surface-sterilized sclerotia of mutants and strain 1980 of *S. sclerotiorum* were submerged into the conidial suspension of *C. minitans* for 30 min, half-buried in sterilized wet sand in plates, and incubated for 30 days at 20 °C. Twenty sclerotia with five repeats were used for each strain, and the experiment was repeated twice independently. The rot index of sclerotia was evaluated according to the described method [41]. The significance level is *p* < 0.01 and the significant differences are distinguished by a capital letter at the top of bar chart.

When coating sclerotia of *S. sclerotiorum* with conidia, mycelia of *C. minitans* appeared 5 to 7 days earlier on the silencing mutants than on strain 1980 (Figure 5a). Approximately 30% germinated conidia were detected in the sclerotial soak of strain 1980, while an average of 45% conidia germinated in the sclerotial soak of the *SsNEP2* silencing mutants (Figure 5b). Sclerotia of all strains were parasitized after 30 days of inoculation, and pycnidia were formed on the surface of the parasitized sclerotia (Figure 5c). The rot index of silencing mutants was much less than that of the wild strain 1980 (Figure 5d). These results demonstrate that silencing of *SsNEP2* in *S. sclerotiorum* promotes conidial germination and enhances resistance to the parasitism of *C. minitans*.

### 3.5. Overexpression of SsNEP2 Promotes the Growth of C. minitans

**Figure 6:**
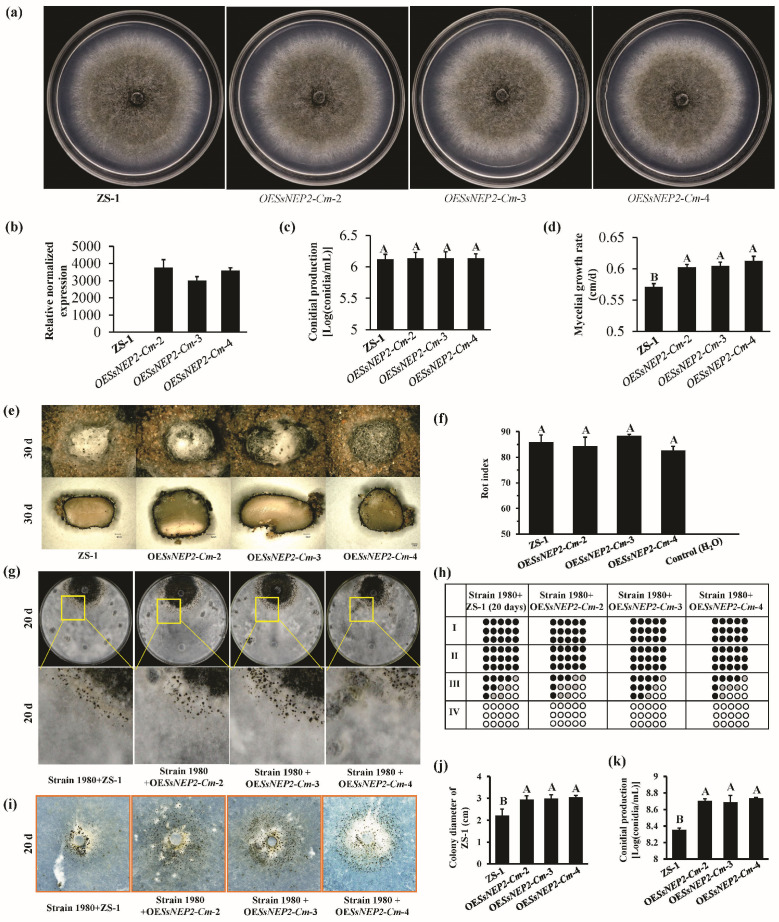
*SsNEP2* overexpressed mutants of *C. minitans*. (**a**) Colony morphology of *SsNEP2* overexpressed mutants of *C. minitans* OE*SsNep2*-*Cm*-2, OE*SsNep2*-*Cm*-3, and OE*SsNep2*-*Cm*-4. (**b**) qPCR detection of the expression of *SsNEP2* in *C. minitans*. (**c**) Conidial production of *SsNEP2* overexpressed mutants of *C. minitans*. (**d**) Growth rate of the *SsNEP2* overexpressed mutants of *C. minitans*. (**e**,**f**) Sclerotial parasitism of the *SsNEP2* overexpressed mutants of *C. minitans*. Sclerotia are from the *S. sclerotiorum* strain 1980. ZS-1 represents the *C. minitans* strain ZS-1. (**g**,**h**) Mycelium parasitism of *SsNEP2* overexpressed mutants of *C. minitans* on PDA. Each circle represents a colony developed from a mycelial agar disk sampled from zones I, II, III, or IV between the inoculation sites of *C. minitans* and *S. sclerotiorum* in a dual culture. (**i**) Mycelial parasitism of *SsNEP2* overexpressed mutants of *C. minitans* on water agar. (**j**) The colony diameter of *C. minitans* from the samples of (**e**). (**k**) The conidial production of *C. minitans* from the samples of (**e**). The significance level is *p* < 0.01 and the significant differences are distinguished by a capital letter at the top of bar chart.

As a secreted protein-encoding gene, the expression of *SsNEP2* was induced during the interaction between *S. sclerotiorum* and *C. minitans*. The resistance to parasitism by *C. minitans* was enhanced by the silencing mutants. It is postulated that *SsNEP2* may enhance the parasitic ability and biocontrol potential of *C. minitans* on *S. sclerotiorum*. To further explore the parasitic effect on *C. minitans*, *SsNEP2* was introduced into strain ZS-1. Three *SsNEP2* overexpressed mutants in *C. minitans* (OE*SsNep2*-*Cm*-2, OE*SsNep2*-*Cm*-3, and OE*SsNep2*-*Cm*-4) were obtained (Figure 6a,b). There was no significant difference in the colony morphology and conidiation of *SsNEP2* overexpressed mutants compared to those of strain ZS-1 on PDA (Figure 6c). The average mycelial growth rate of three overexpressed mutants, namely OE*SsNep2*-*Cm*-2, OE*SsNep2*-*Cm*-3, and OE*SsNep2*-*Cm*-4, was 0.60 cm/d, 0.60 cm/d, and 0.61 cm/d, respectively, which was significantly higher than that of strain ZS-1 (0.57 cm/d) (Figure 6d).

### 3.6. Expression of SsNEP2 in C. minitans Improves Parasitism to Mycelia of S. sclerotiorum

Three overexpressed mutants in *C. minitans*, namely OE*SsNep2*-*Cm*-2, OE*SsNep2*-*Cm*-3, and OE*SsNep2*-*Cm*-4, were used to explore the function of *SsNEP2* during the mycoparasitic process. A similar rot index of sclerotia and parasitism to the mycelia of *S. sclerotiorum* by dual culture was observed among all strains of *C. minitans* cultured on PDA (Figure 6e–h). However, when growing on the colony of *S. sclerotiorum* for 20 days, the average colony diameter of OE*SsNEP2*-*Cm*-2, OE*SsNEP2*-*Cm*-3, and OE*SsNEP2*-*Cm*-4 was 2.94 cm, 2.99 cm, and 3.05 cm, which was significantly larger than that of strain ZS-1 (2.22 cm) (Figure 6i,j), and more conidia were formed by overexpressed mutants of *C. minitans* than strain ZS-1 (Figure 6k). Therefore, the overexpression of *SsNEP2* in *C. minitans* improves mycoparasitic ability against mycelia of *S. sclerotiorum* and conidial production there.

### 3.7. Expression of SsNEP2 in C. minitans Restores Conidiation and Parasitism to Silencing Mutants of S. sclerotiorum

**Figure 7:**
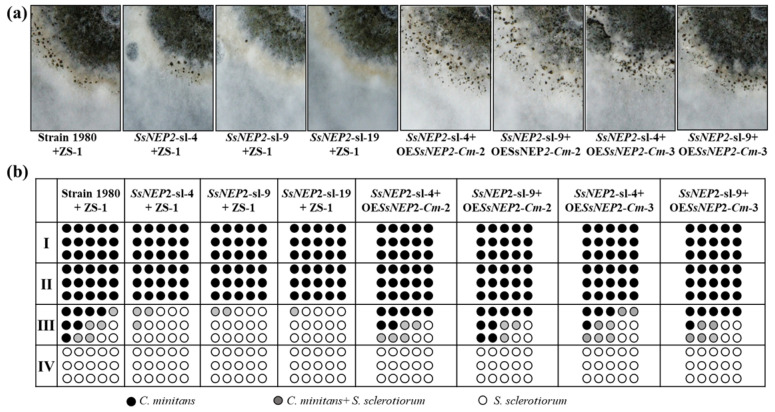
Mycelial parasitism between *SsNEP2* silencing transformants of *S. sclerotiorum* and expression mutants of *C. minitans.* (**a**) Dual culture of *C. minitans* and *S. sclerotiorum* for 20 days under 20 °C. ZS-1 represents the *C. minitans* strain ZS-1. (**b**) Results of the dual culture of (**a**). Schematic diagram showing the number of agar disks that gave rise to either *C. minitans* (black circle), *S. sclerotiorum* (hollow circle), or both (gray circle). Each circle represents a colony developed from a mycelial agar disk sampled from zones I, II, III, or IV between the inoculation sites of *C. minitans* and *S. sclerotiorum* in a dual culture.

To further evaluate the role of *SsNEP2* in mycoparasitism, two *SsNEP2* overexpressed mutants of *C. minitans*, namely OE*SsNep2*-*Cm*-2 and OE*SsNep2*-*Cm*-3, were dual cultured with the silencing mutants *SsNEP2*-sl-3 and *SsNEP2*-sl-9, respectively. The parasitism of OE*SsNep2*-*Cm*-2 and OE*SsNep2*-*Cm*-3 to *SsNEP2*-sl-3 and *SsNEP2*-sl-9 was much stronger than ZS-1, and they also produced more pycnidia (Figure 7). The results indicate that the overexpression of *SsNEP2* in *C. minitans* restores the effects on mycoparasitic ability and conidial production by the silencing of *SsNEP2* in *S. sclerotiorum* during the interaction process.

## 4. Discussion

NLP expression was significantly up-regulated at the early stage of infection between the pathogens and their hosts, and the role of NLPs was diversified during infection [18,33,42,43,44]. Most NLPs played dual roles in plant–pathogen interactions as toxin-like virulence factors and as triggers of plant innate immune responses, inducing plant necrosis [15,18,27,45,46,47,48,49]. NPP1 in plant leaves resulted in transcript accumulation of pathogenesis-related (PR) genes, production of ROS and ethylene, callose apposition, and HR-like cell death [14,19,32,42]. The synthesized peptides of *Valsa mali* VmNLP2 triggered a strong immune response in *Arabidopsis thaliana* and induced cell death when transiently expressed in *Nicotiana benthamiana* [50]. In *Theobroma cacao*, necrosis was observed on leaves after NEP1 treatment, and expression of the defense-related gene *TcWRKY-1* was enhanced, while expression of *TcChiB*, *TcLhca-1,* and *TcrbcS* was repressed [51]. Interestingly, seven *NLPs* were strikingly up-regulated during the early stages of the infection process between *Plasmopara viticola* and grapevine leaves, while only PvNLP7 could induce necrosis and disease resistance in *N. benthamiana* by *A. tumefaciens*-mediated transient expression [52]. When there was infiltration of recombinant NLPs into tobacco and *Arabidopsis* leaves, none of the 12 identified HaNLPs in *Hyaloperonospora arabidopsidis* was able to induce necrosis [53]. Meanwhile, significantly reduced virulence on leaves of tobacco or *Arabidopsis* and decreased accumulation of ROS were reported in the knock-out of *SsNEP2* in *S. sclerotiorum* [33]. The disruption of NLP in *Stemphylium lycopersici* led to low conidial production, weak effects on its adaptation to oxidative stress, and significant reduction in its virulence on tomato [54]. However, as described in the Introduction section, in most plant pathogens, the absence of NLPs did not affect their pathogenicity [21,22,28,30,43]. These suggest that NLPs might be functional as PAMPs in inducing resistance and necrosis but not as a major virulence determinant during most infection systems of fungi and their host plants.

NLPs are widely present in fungi with diverse functions, e.g., induction of ROS accumulation and involvement in host selection. In *S. sclerotiorum*, *SsNEP2*, encoding a putative secreted effector, was up-regulated at 24 h (log FC = 5.17) after inoculation on plants [55] and was reported to cause necrosis on tobacco leaves [23]. Further study showed that SsNEP2 served as a PAMP to trigger immunity of plants, and the loss of *SsNEP2* decreased the virulence to tobacco or *Arabidopsis* [33]. Interestingly, when coming in contact with *C. minitans* for 12 hpi, *SsNEP2* was also significantly up-regulated (log FC = 4.91) in *S. sclerotiorum* [11] and up-regulated with log FC = 9.88 for 24 hpi. Silencing of *SsNEP2* led to enhanced resistance to parasitism of *C. minitans*. In this study, an additional function of *SsNEP2* was explored in *S. sclerotiorum* during interaction with the mycoparasite *C. minitans*.

The parasitism of *C. minitans* against *S. sclerotiorum* is a comprehensive and complex process. Several factors in *C. minitans* that reduce the mycoparasitism ability have been analyzed, including cyclic GMP (cGMP), cyclic AMP (cAMP), mitogen-activated protein (MAP) kinase cascade, peroxisome biogenesis gene (*CmPEX6*) [8], NADPH oxidase complex, autophagy, PIF1 DNA helicase gene [56], homotypic fusion and vacuolar protein-sorting gene (CmVps39) [57], oxalate decarboxylase-coding genes [9,58], altered inheritance of mitochondria protein 24 (*CmAim24*) [59], and melanin synthesis-related gene 1 (*CmMR1*) [60], etc. However, research on *S. sclerotiorum* and the activation of mycoparasitism remains rare. Silencing of *SsNEP2*, which enhanced the resistance to parasitism of *C. minitans*, could be a method by which parasitic ability is not activated. The first layer of the plant surveillance system invokes the recognition of microbes by employing pattern recognition receptors [61,62,63]. A class of NLPs has been described as PAMPs from mostly phytopathogens [15,16,47]. NLPs are cytotoxic to eudicot plants, as they disturb the plant plasma membrane by binding to specific plant membrane sphingolipid receptors [64]. *SsNEP2* expressed in *C. minitans* improved mycelial parasitism on *S. sclerotiorum* and restored the parasitic ability affected by the silencing of *SsNEP2* of *S. sclerotiorum*, while the silencing of *SsNEP2* in *S. sclerotiorum* inhibited the production of *C. minitans* conidia but promoted their germination. Therefore, it could be concluded that *SsNEP2* might also act as a PAMP to be recognized by *C. minitans* and further activate the parasitic system between *S. sclerotiorum* and *C. minitans*. As a specific parasitic fungus of *S. sclerotiorum*, *C. minitans* is completely non-pathogenic to plants, and its biocontrol value has been verified [6,65]. *SsNEP2* can be applied to enhance the biological potential of *C. minitans*.

## Figures and Tables

**Table 1:** Primers involved this research.

Primer Name	Sequence (5′ to 3′)	Function
*SsNEP2*-*Eco*RV R	GATATCCCTCGATGTCGTAAATGGCTGC	*SsNEP2* sense fragment to construct a silencing vector with *Eco*RV and *Cla*I restriction enzyme cleavage sites
*SsNEP2*-*Cla*I F	CCATCGATATTCTTGAACGGAACATTCGCCT	
*SsNEP2*-*Sma*I F	TCCCCCGGGCCTCGATGTCGTAAATGGCTGC	*SsNEP2* antisense fragment to construct a silencing vector with *Sma*I and *Bam*HI restriction enzyme cleavage sites
*SsNEP2*-*Bam*HI R	CGGGATCCATTCTTGAACGGAACATTCGCCT	
OEs*sNEP2*- SpeI F	ACCTTCAAAGAGCTCACTAGTATGGTTGCCTTTGCCAAATC	The full-length coding sequence of *SsNEP2* to construct an overexpressing vector with *Spe*I and *Kpn*I restriction endonuclease cleavage sites
OEs*sNEP2*-KpnI R	GTAGTCCATCCCGGGGGTACCGAAACTACTAGCCTTCACAAAGTTATTCT	
Rt*SsNep1*-F	CTTTGGGAAGAGATTTACC	Expression of *SsNEP1* in *S. sclerotiorum*
Rt*SsNep1*-R	GTTGAATGGACAGTTAGC	
Rt*SsNep2*-F	ATCATTCCTGTGGTCTTAC	Expression of *SsNEP2* in *S. sclerotiorum*
Rt*SsNep2*-R	AATCCGTATTCTCAAGCG	
*CmActin* F	GATTGGTATGGGTCAGAA	Expression of *actin* in *C. minitans*
*CmActin* R	ATCTGGGTCATCTTCTCA	
*β-tubulin* F	TTGGATTTGCTCCTTTGACCAG	Expression of *β-tubulin* in *S. sclerotiorum*
*β-tubulin* R	AGCGGCCATCATGTTCTTAGG	

## Data Availability

The original contributions presented in this study are included in the article. Further inquiries can be directed to the corresponding author.

## Abbreviations

The following abbreviations are used in this manuscript:

NEP1	necrosis- and ethylene-inducing peptide 1
NLP	NEP1-like protein
NPP1	The necrosis-inducing protein
PAMP	pathogen-associated molecular patterns
ROS	regulating reactive oxygen species
hpi	hours post-inoculation
PDA	potato dextrose agar plates
suc2	secretion-defective invertase gene
CMD-W	a medium lacking tryptophan, 0.67% yeast N base without amino acid, 0.075% W dropout supplement, 2%sucrose, 0.1% glucose, and 2% agar
YPRAA	a medium containing 10 g/L yeast extract, 20 g/L peptone, 20 g/L raffinose, 2 mg/L antimycin A, and 2% agar
TTC	2,3,5-triphenyltetrazolium chloride
ATMT	*Agrobacterium tumefaciens*-mediated transformation
STC	a kind of medium containing 1.0 mol/L sorbitol, 0.05 mol/L Tris-HCl pH 8.0, and 0.05 mol/L CaCl2
RT-PCR	reverse transcription PCR
RT-qPCR	quantitative reverse transcription PCR
ddH_2_O	double distilled water
FPDB	PDB fermentation broth
PDA-FPDB	filtered FPDB amended with PDA at 1:1 volume
WA-FPDB	filtered FPDB amended with water containing 2% agar at 1:1 volume
